# Quantifying cup overhang after total hip arthroplasty: standardized measurement using reformatted computed tomography and association of overhang distance with iliopsoas impingement

**DOI:** 10.1007/s00330-023-10479-5

**Published:** 2023-12-26

**Authors:** Adrian A. Marth, Christian Ofner, Patrick O. Zingg, Reto Sutter

**Affiliations:** 1 Swiss Center for Musculoskeletal Imaging, Balgrist Campus AG, Zurich, Switzerland; 2 Department of Radiology, Balgrist University Hospital, Faculty of Medicine, University of Zurich, Forchstrasse 340, 8008 Zurich, Switzerland; 3 Department of Orthopaedics, Balgrist University Hospital, University of Zurich, Zurich, Switzerland

**Keywords:** Arthroplasty (replacement, hip), Pain (postoperative), Impingement, Tomography (x-ray computed)

## Abstract

**Objectives:**

Currently, there is no standardized measurement method for evaluating cup overhang (CO) in patients undergoing total hip arthroplasty (THA). We propose a novel, standardized method of measuring overhang distance in patients following primary total hip arthroplasty (THA) on computed tomography (CT) images after multiplanar reformation and compare it to a previously proposed measurement method on sagittal CT images.

**Materials and methods:**

This retrospective study included patients who underwent primary THA with an anterior approach. Patients with cup overhang (CO) and iliopsoas impingement (IPI) were identified by clinical and imaging data. Uncorrected overhang distance (OD_u_) was evaluated on orthogonal sagittal CT images while corrected overhang distance (OD_c_) was evaluated on reformatted sagittal CT images corrected for pelvic rotation and aligned with the plane of the cup face.

**Results:**

Out of 220 patients with THA, 23 patients (10.4%) with CO and 16 patients (7.3%) with IPI were identified. CO was significantly associated to IPI (*p* < 0.001). The inter- and intrareader agreement was almost perfect for OD_c_ (*κ* = 0.822, *κ* = 0.850), whereas it was fair and moderate for OD_u_ (*κ* = 0.391, *κ* = 0.455), respectively. The discriminative ability of OD_c_ was excellent (area under the curve (AUC) = 0.909 (95% confidence interval (CI) 0.784–1.000)) in the receiver operating characteristic analysis. Conversely, AUC for OD_u_ was poor, measuring 0.677 (95% CI 0.433–0.921).

**Conclusion:**

We implemented a novel measurement method for CT images reformatted at the plane of the cup face to assess overhang distance in patients with CO following THA.

**Clinical relevance statement:**

While further validation is necessary, the proposed method is characterized by its high reproducibility and might be used to predict the occurrence of iliopsoas impingement in patients with cup overhang following total hip arthroplasty.

**Key Points:**

• *A novel, standardized method of measuring cup overhang distance in patients following primary total hip arthroplasty on CT images is proposed.*

• *Cup overhang was associated to iliopsoas impingement. The proposed method was reproducible and showed excellent prediction of iliopsoas impingement in patients with cup overhang.*

• *This method can be implemented in clinical practice when assessing CT images of patients with cup overhang for iliopsoas impingement.*

**Supplementary Information:**

The online version contains supplementary material available at 10.1007/s00330-023-10479-5.

## Introduction

Primary total hip arthroplasty (THA) is the most common joint replacement, with 625,000 prostheses implanted in the USA between 2012 and 2019 [1]. While most THA have an excellent outcome, iliopsoas impingement (IPI) is an underrecognized cause of persistent groin pain in THA patients, with an incidence of approximately 2–7% [2–4]. Cup overhang (CO) is visible on computed tomography (CT) scans as overlap of the THA cup beyond the bony surface of the acetabulum [5] and can lead to iliopsoas tendon impingement and mechanical tendon irritation during hip flexion. CO can arise from oversized cups or a version mismatch between the cup and the native acetabulum of the patient [6–9] and has been identified as a risk factor for IPI, even though there is no consensus at which overhang distance patients become symptomatic [5, 7, 9–12]. Other suspected risk factors of IPI include female sex, leg length discrepancy, and retained cement or screw protrusion [13–15]. The diagnosis of IPI is made by analyzing the patient’s medical history, conducting a physical examination, and reviewing plain radiographs or computed tomography (CT) scans [2]. Additionally, ultrasonography or magnetic resonance imaging (MRI) can be employed to assess the presence of iliopsoas tendonitis [16].

Currently, there is no standardized method for precisely measuring overhang distance (OD) in patients with CO. Even though one study has shown the impact of pelvic tilt on OD measurements [17], all other studies that attempted to measure OD on non-reformatted CT images or plain radiographs have not yet taken the impact of acetabular inclination as well as pelvic rotation into account [7–9, 11, 18, 19].

Therefore, the purpose of this study was to propose a reproducible method for measuring OD in CT images after multiplanar reformation (MPR) and to compare it to a previously proposed measurement method on orthogonal sagittal images. Additionally, we aimed to evaluate the impact of the measured OD on the occurrence of iliopsoas impingement in patients undergoing primary THA.

## Materials and methods

### Patient selection

This single-center, retrospective study was approved by the local ethics committee (Cantonal ethics committee Zurich). All procedures conducted in this study adhered to the ethical standards established by the institutional and/or national research committee, in accordance with the principles outlined in the 1964 Helsinki Declaration and its subsequent amendments, or other comparable ethical guidelines.

**Fig. 1:**
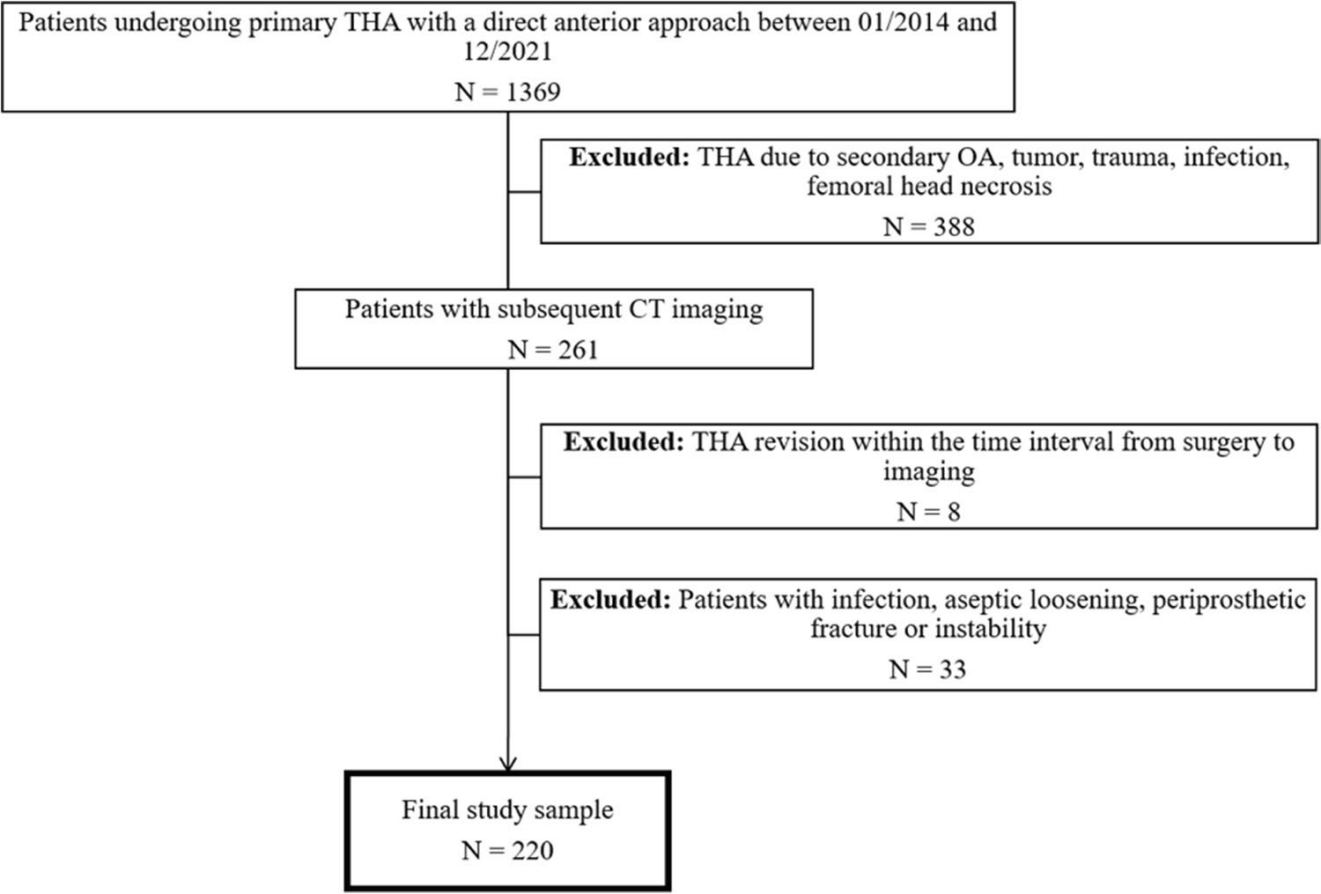
Flowchart depicting the patient selection process. THA, total hip arthroplasty; OA, osteoarthritis

To identify patients for our study, a query of the hospital’s information system retrieved records of all THAs performed with a direct anterior approach between January 2014 and December 2021 by various surgeons within our institution (Fig. 1). Prostheses were made of either cobalt-chromium-molybdenum alloy or titanium alloy. Patients who received THA due to secondary osteoarthritis (e.g., hip dysplasia), tumor, trauma, infection, and femoral head necrosis or patients who had a history of THA revision were excluded from this study. Subsequently, we accessed the radiology information system to identify patients who had undergone CT examinations of the pelvic region after surgery. CT was performed for complications after THA (118/220; 53.6%), for postoperative assessment of positioning of THA components (45/220; 20.5%), or for other clinical indications (57/220; 25.9%). All examinations were performed with spectral shaping (tin prefiltration) according to the local clinical standard. After applying these criteria, CT examinations of 261 patients were identified for further analysis. Patients with THA revision within the time interval from surgery to imaging as well as documented infection, aseptic loosening, periprosthetic fracture, or instability as reason for groin pain were excluded. This led to a final sample size of 220 patients. We assessed surgical or clinical reports and imaging data (magnetic resonance imaging and ultrasound) of this sample to identify documented cases of IPI. IPI diagnosis followed a clinical procedure performed by orthopedic surgeons. Firstly, a detailed history was taken in patients admitted for hip pain after THA, which included inquiring about the onset of symptoms, the location of the pain, and activities or movements that alleviated or aggravated the pain. Next, a physical examination was conducted to evaluate the range of motion, including palpation for areas of tenderness or muscle tightness. Additionally, specific clinical assessments for iliopsoas tightness, such as the Thomas Test, were conducted. Diagnosis of IPI was confirmed only if imaging supported the clinical diagnosis, which was defined as the presence of either iliopectineal bursitis or iliopsoas tendinopathy on MR or ultrasound images [2]. In a subgroup of patients, diagnosis was confirmed and treated through fluoroscopy-guided corticosteroid injections. CO on CT images was considered confirmed if the cup extended beyond the bony surface of the acetabulum.

### Image analysis

CT scans were acquired on 64-slice and 128-slice CT scanners (Edge Plus, or Definition AS Plus; Siemens Healthineers, or Brilliance 64; Philips Healthcare) at 120–140 kVp for different clinical indications. Eighty-four out of the 220 CT examinations (38.2%) were performed with tin prefiltration. Slice thicknesses ranged from 1 to 2 mm. Two fellowship-trained radiologists (A.A.M., C.O.) analyzed the images for CO and independently performed OD measurements. Additionally, the first reader repeated the OD measurements after 3 months to assess intrareader agreement. Both readers were blinded to demographic and clinical data and analyzed the images independently and in random order on the local Picture Archiving and Communications (PACS) viewer (Merlin, Phoenix-PACS GmbH).

**Fig. 2:**
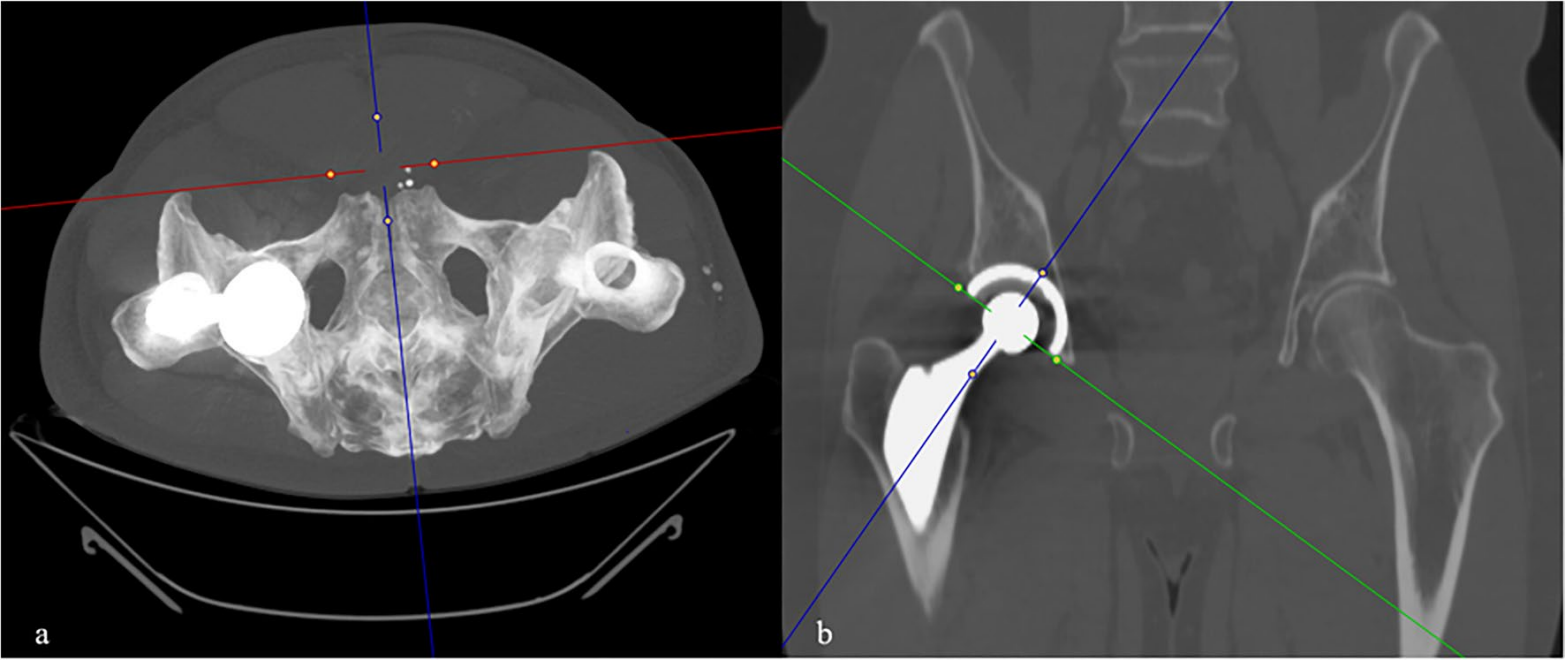
Multiplanar reformation of CT scans for corrected overhang distance measurement. Maximum intensity projection in the axial plane along the anterior superior iliac spine to correct for pelvic rotation (**a**) and angulation in the coronal plane along the plane of the cup face (**b**). The corrected overhang distance (OD_c_) is then measured on the corrected sagittal images parallel to the blue MPR reference line, as shown in Fig. 3. MPR, multiplanar reformation

**Fig. 3:**
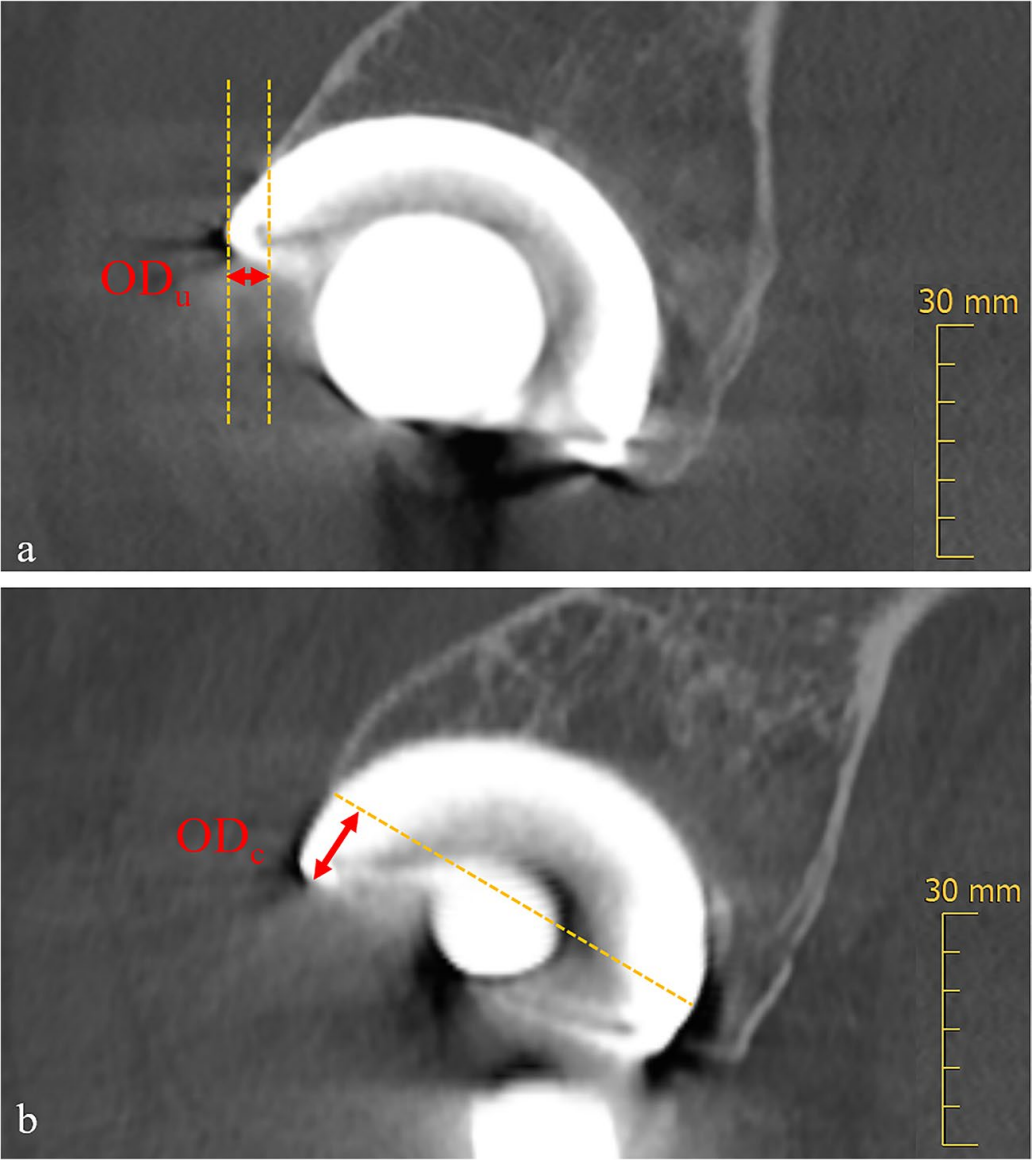
The orthogonal sagittal CT image of a patient with cup overhang without multiplanar reformation (MPR): This uncorrected overhang distance (double arrows, OD_u_) is measured according to the current literature (**a**). After MPR-aided correction for pelvic rotation and with coronal MPR angled at the plane of the cup face (**b**), the measurement of the overhang distance perpendicular to the cup circumference is shown in the same patient (double arrows, OD_c_). All measurements were carried out at the slice in which the maximum distance of uncovered cup was visible

For measuring MPR-corrected OD (OD_c_), the following approach was conducted: First, a MPR of the CT scans was performed aided by maximum intensity projection (Fig. 2). In the axial plane, images were adjusted along the anterior superior iliac spine to account for pelvic rotation [20]. Next, the coronal MPR was angled along the plane of the cup face. Afterwards, the measurement was conducted by determining the distance from a line perpendicular to the cup circumference, originating from the point of which the anterior cup surface is exposed from the bone (Fig. 3). For the uncorrected measurement of OD (OD_u_), the anteroposterior distance of uncovered cup was measured on orthogonal sagittal images. All distances were measured in millimeters at the slice in which the maximum distance of the uncovered cup was visible. Both readers were allowed to adjust window levels individually to assess the boundary of the bone and to minimize metal artifacts. Additionally, the time for MPR preparation and measurement of OD_c_ was timed with a stopwatch for both readers.

### Statistical analysis

The statistical analyses were conducted using SPSS Statistics (v25, IBM Corp.). Normal distribution and homogeneity of variances for continuous variables were confirmed using the Shapiro–Wilk test and Levene’s test, respectively. Group differences were evaluated with Student’s *t* test in case of normal distribution and with the Mann–Whitney *U* test in case of non-normal distribution, while the chi-square analysis was conducted for categorical variables. Interreader and intrareader agreement was assessed by a kappa statistic (Cohen’s kappa). Receiver operating characteristic (ROC) curves were used to measure the predictive accuracy (area under the curve, AUC) for both measurement methods. AUC values > 0.9 were classified as “excellent” and values < 0.7 as “poor” [21]. The Youden index was used to aid with the selection of the optimal sensitivity and specificity threshold. *p* values < 0.05 were considered statistically significant.

## Results

### Patient characteristics, cup overhang, and iliopsoas impingement

**Fig. 4:**
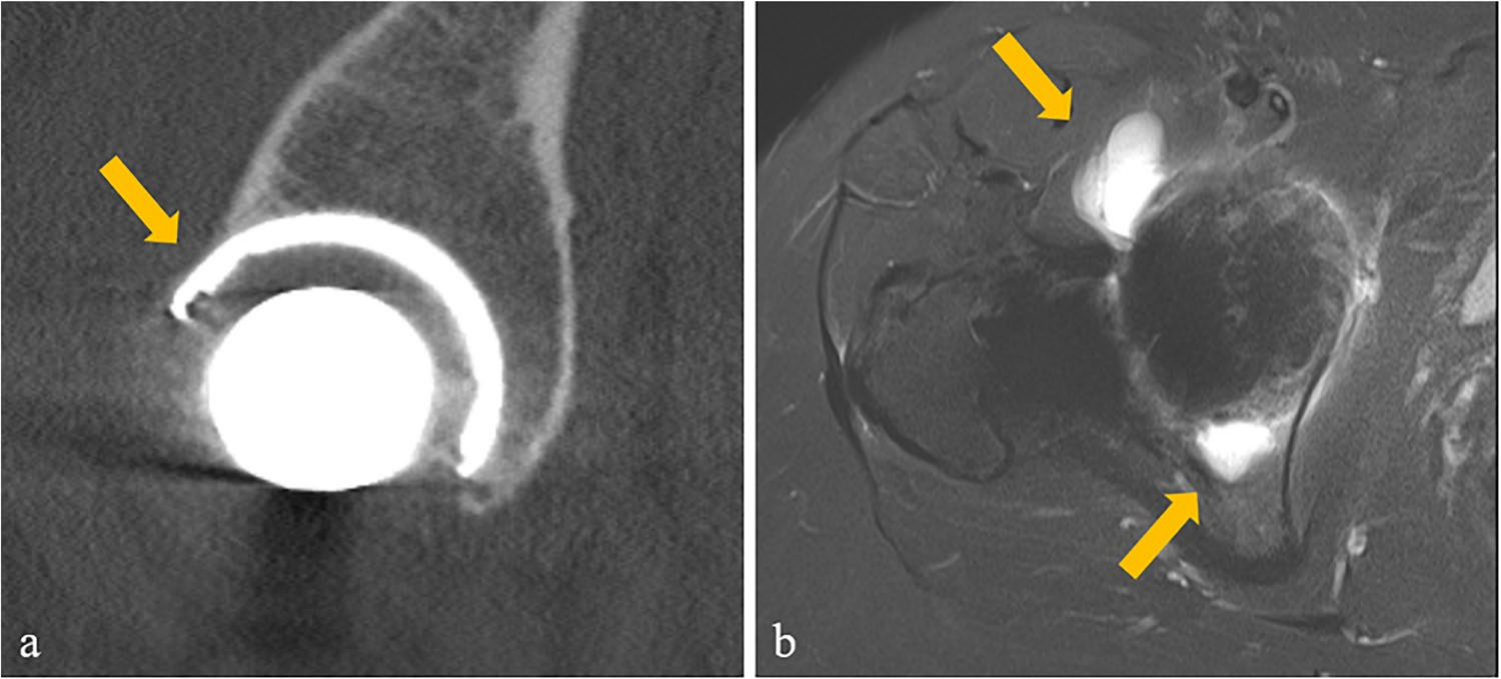
Exemplary images of a 64-year-old female patient with cup overhang and clinically diagnosed iliopsoas impingement following total hip arthroplasty. Sagittal CT images (**a**) clearly depicting the cup overhang. Axial STIR images of the right hip depicting fluid in the bursa iliopectinea as a sign of mechanical irritation of the iliopsoas tendon by cup overhang (**b**). STIR, short-tau inversion recovery

Mean age at surgery was 62.7 ± 12.7 years and mean time from surgery to CT imaging was 390 ± 620 days (Table 1). IPI was documented in the clinical notes in 16 patients (7.3%), while 23 patients (10.4%) revealed CO on CT imaging (Tables 1 and 2). IPI was treated with corticosteroid injection in five patients (31.3%) and with iliopsoas tenotomy in three patients (18.8%), while eight patients (50.0%) were treated conservatively. An exemplary image of a patient with IPI and CO is depicted in Fig. 4. The Mann–Whitney *U* test revealed no significant differences for age, BMI, and time from surgery to CT imaging between the CO group (*n* = 23) and the non-CO group (*n* = 197) as well as between the IPI group (*n* = 16) and the non-IPI group (*n* = 204) (all *p* ≥ 0.242, Table 1). The chi-square analysis revealed a significant association of CO to IPI (*p* < 0.001, OR 35.20, 95% confidence interval (CI) 10.53–117.73). However, no significant association was found between sex and IPI (*p* = 0.205, OR 1.95, 95% CI 0.68–5.57).

**Table 1:** Characteristics of the total study population and of the subgroups with and without cup overhang

	Total study population (*n* = 220)	Cup overhang (*n* = 23)	No cup overhang (*n* = 197)	*p* value
Age (years)	62.7 ± 12.7	62.5 ± 12.8	62.7 ± 12.7	0.942
Sex (female, %)	52.7	52.8	52.2	0.955
BMI (kg/m^2^)	28.1 ± 5.7	29.4 ± 6.8	27.9 ± 5.6	0.242
Time from surgery to imaging (days)	389 ± 621	472 ± 667	380 ± 616	0.501
Iliopsoas impingement (%)	7.3	47.8	2.5	< 0.001*
Mean overhang distance (mm)				
Uncorrected method		6.0 ± 2.8		
MPR-corrected method		10.2 ± 4.1		

*BMI*, body mass index; *MPR*, multiplanar reformation

^*^Statistical significance

**Table 2:** Characteristics of the subgroups with and without iliopsoas impingement (IPI)

	IPI (*n* = 16)	No IPI (*n* = 204)	*p* value	OR (95% CI)
Age (years)	64.1 ± 11.5	62.6 ± 12.8	0.648	
Sex (female, %)	62.5	46.1	0.205	1.950 (0.683–5.567)
BMI (kg/m^2^)	28.3 ± 5.8	28.0 ± 5.7	0.877	
Time from surgery to imaging (days)	611 ± 654	371 ± 616	0.137	
Cup overhang (%)	68.8	5.9	< 0.001*	35.200 (10.525–117.728)
Mean overhang distance (mm)				
Uncorrected method	6.7 ± 2.9	5.1 ± 2.6	0.364	1.119 (0.810–1.775)
MPR-corrected method	12.5 ± 3.6	7.4 ± 2.5	0.002*	5.327 (0.809–35.098)

*BMI*, body mass index; *CI*, confidence interval; *MPR*, multiplanar reformation; *OR*, odds ratio

^*^Statistical significance

### Results for corrected and uncorrected OD measurements

**Fig. 5:**
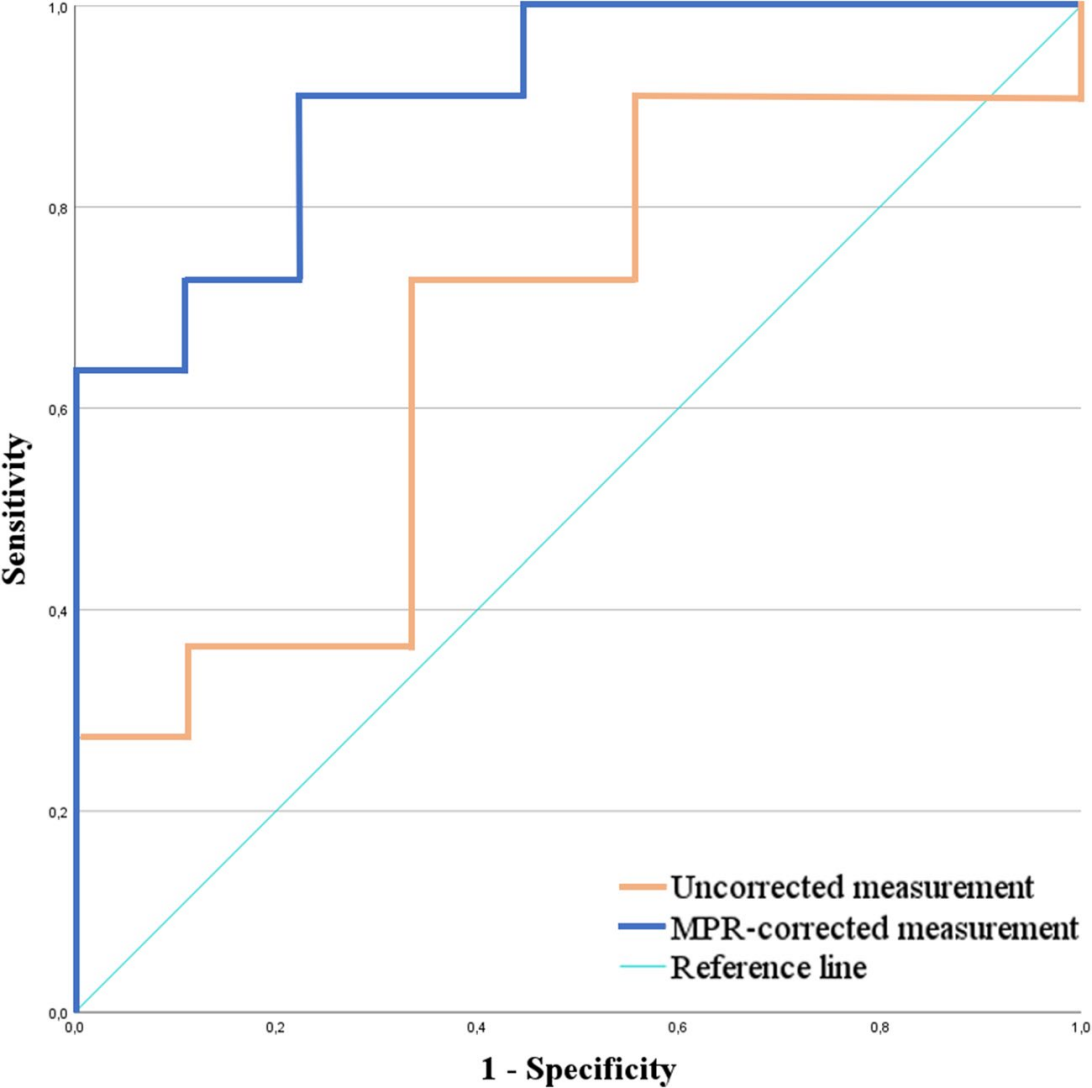
Receiver operating characteristic curve for both overhang distance measure methods. Predictive accuracy for the presence of IPI was excellent for the MPR-corrected method (OD_c_: area under the curve (AUC) = 0.909, 95% CI 0.784–1.000) and poor for the uncorrected measurement method (OD_u_: AUC = 0.677, 95% CI 0.433–0.921). CO, cup overhang; MPR, multiplanar reformation

Mean OD_u_ was 6.0 ± 2.8 mm, while OD_c_ was 10.2 ± 4.1 mm. The interreader agreement was almost perfect for OD_c_ (*κ* = 0.822) and fair for OD_u_ (*κ* = 0.391). The intrareader agreement assessed after a time interval of 3 months was almost perfect for OD_c_ (*κ* = 0.850) and moderate for OD_u_ (*κ* = 0.455). The receiver operating characteristic (ROC) analysis revealed an excellent discrimination for OD_c_ (AUC = 0.909, 95% CI 0.784–1.000) for identifying patients with IPI, whereas the discrimination ability for OD_u_ was poor (AUC = 0.677, 95% CI 0.433–0.921; Fig. 5). The optimal cutoff value for diagnosing cup prominence was 7.5 mm (sensitivity = 90.9%, specificity = 77.8%) for OD_c_, whereas it was 6.4 mm (sensitivity = 80.8%, specificity = 49.1%) for OD_u_. The mean time for MPR preparation and OD_c_ measurement was 10.6 ± 2.1 s.

## Discussion

This study aimed to implement a novel measurement method corrected with multiplanar reformation to assess overhang distance (OD) in patients with cup overhang (CO) following total hip arthroplasty and to compare it with a previously proposed measurement method on orthogonal sagittal CT images.

**Fig. 6:**
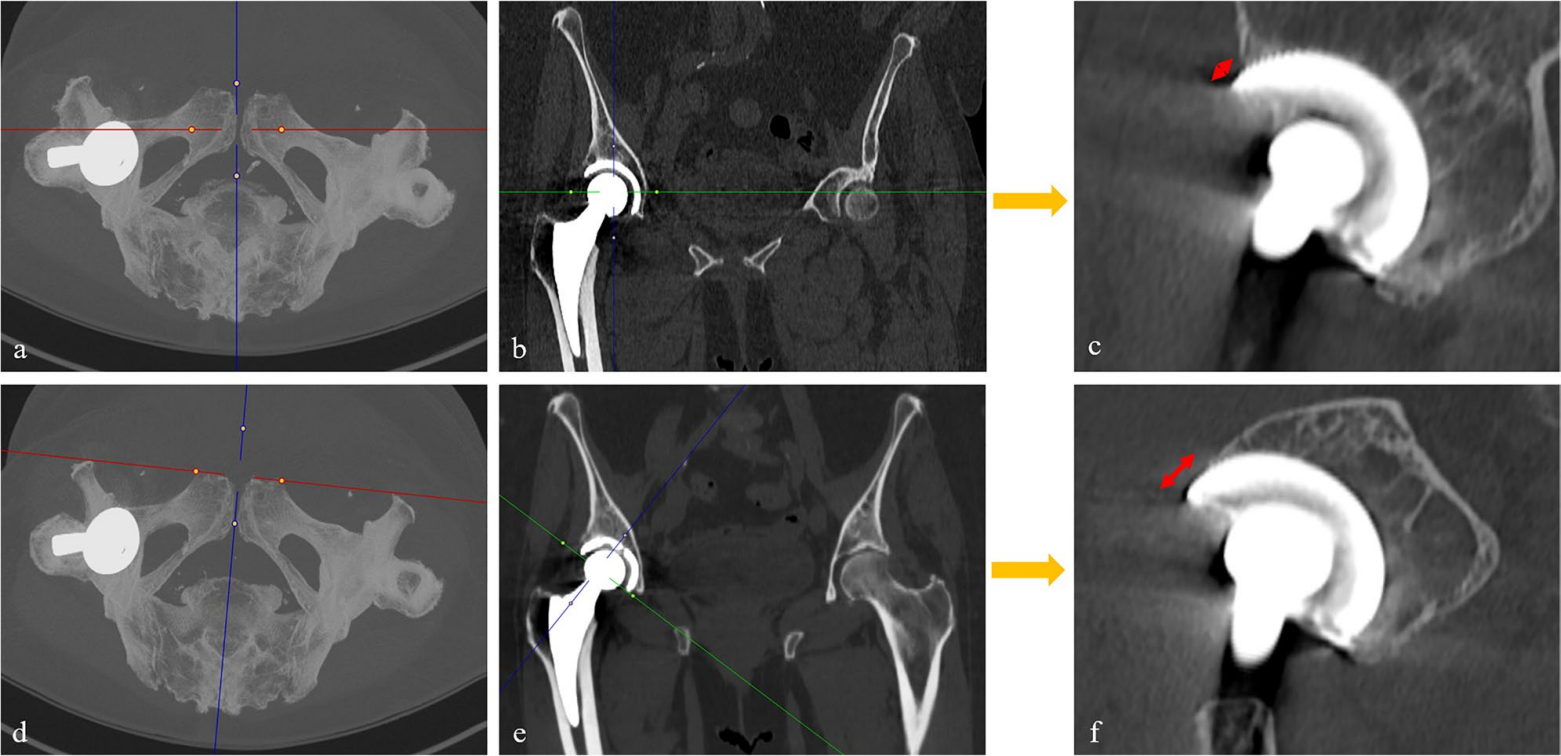
Simulation of overhang distance measurements for didactic purposes (**a**, **b**). Uncorrected standard images, with standard orientation of the MPR reference lines on the axial (**a**) and coronal image (**b**) result in an underestimation of the cup overhang in the sagittal image (**c**), while MPR images corrected for pelvic rotation and angulation at the plane of the cup face (**d**, **e**) allow to measure the corrected overhang distance measurement (OD_c_, **f**). MPR, multiplanar reformation

The first main finding of our study was that the proposed MPR-corrected method to measure OD (OD_c_) demonstrated an excellent inter- and intrareader agreement, corroborating its reliability and high reproducibility. The proposed method is easy to implement in clinical practice, since the MPR adjustment and overhang distance measurement process required only minimal time. On the other hand, uncorrected measurement of OD on orthogonal sagittal images (OD_u_) yielded an inter- and intrareader agreement that was much poorer. One possible explanation for this is the variability of the anterior border of the cup edge, primarily caused by variations in pelvic rotation and cup inclination. Figure 6 shows an example of the impact of different pelvic rotation and cup inclination on the measured OD, exemplified by the use of MPR.

The second main finding of this study was that cup overhang was significantly associated with the presence of IPI and that OD_c_ was excellent at predicting IPI (AUC = 0.909 in the ROC analysis). In addition, the optimal threshold for diagnosing IPI with the corrected method demonstrated an excellent sensitivity, which is higher than in a recent study by Hardwick-Morris et al [12] that followed an approach similar to our uncorrected measurement. This finding is useful for the radiologist when evaluating CT images of patients with suspected IPI and will be valuable for the clinical decision making.

In the present study sample, IPI was observed with a relatively high frequency (7.3%), approaching the upper limit reported in the current literature [2–4]. This might be due to the fact that only patients receiving THA through a direct anterior approach were included in this study. The relationship between this surgical approach and the occurrence of IPI is explained by the disruption of the anterior hip capsule and resulting compromise of the important protective layer between the cup and the iliopsoas complex, possibly leading to tendon irritation [22]. This has also been reported in a study by Dora et al, in which the authors described that the iliopsoas muscle was seen through a defect of the neocapsule at the level of the uncovered cup [10].

Moreover, the present study found that female sex increased the risk of IPI by an odds ratio (OR) of 1.95, even though this finding was non-significant (*p* = 0.205). We believe that the lack of significance might be due to the small size of the IPI group, as in the current literature, the association between female sex and IPI has already been described [18]. This relationship is attributed to the smaller native acetabular diameters typically observed in women, which might constrain the surgeon to enlarge the acetabulum to insert a larger prosthetic femoral head, thus increasing the risk of cup overfitting [22, 23].

We acknowledge several limitations of the study. First, the retrospective design might have led to a selection bias and influenced the observed results. However, we believe that only including patients that underwent THA through a direct anterior approach and excluding patients with secondary osteoarthritis (e.g., due to hip dysplasia) strengthen the internal validity of our findings. Second, the sample size of this study was small, which may limit the generalizability of the findings. Moreover, we could only adjust for sex as a confounding variable when investigating the relationship between IPI, CO, and OD. Furthermore, given the novelty of the proposed MPR-corrected method for assessing OD following THA, we could not validate the reported measurement results with the existing literature.

In summary, this study implemented a novel, MPR-corrected measurement method to assess OD in patients with CO following THA. While further validation of the proposed method is necessary, our results indicate that it is highly reproducible and can be used to identify IPI in patients with CO and THA.

### Supplementary Information

Below is the link to the electronic supplementary material.

Supplementary file1 (RAR 2749 KB)

## Abbreviations

AUC: Area under the curve
CI: Confidence interval
CO: Cup overhang
IPI: Iliopsoas impingement
OD: Overhang distance
OD_c_: Corrected overhang distance
OD_u_: Uncorrected overhang distance
ROC: Receiver operating characteristic
THA: Total hip arthroplasty

## Declarations

### Guarantor

The scientific guarantor of this publication is Reto Sutter, MD.

### Statistics and biometry

No complex statistical methods were necessary for this paper.

### Study subjects or cohorts overlap

No.

### Methodology

• retrospective

• observational

• performed at one institution
